# Trends in sexually transmitted infections in the Netherlands, combining surveillance data from general practices and sexually transmitted infection centers

**DOI:** 10.1186/1471-2296-11-39

**Published:** 2010-05-20

**Authors:** Ingrid VF van den Broek, Robert A Verheij, Christel E van Dijk, Femke DH Koedijk, Marianne AB van der Sande, Jan EAM van Bergen

**Affiliations:** 1 Centre for Infectious Disease Control, RIVM National Institute of Public Health and the Environment, Bilthoven, The Netherlands; 2 NIVEL Netherlands Institute for Health Services Research, Utrecht, The Netherlands; 3 STI AIDS Netherlands, Amsterdam, The Netherlands

## Abstract

**Background:**

Sexually transmitted infections (STI) care in the Netherlands is primarily provided by general practitioners (GPs) and specialized STI centers. STI surveillance is based on data from STI centers, which show increasing numbers of clients. Data from a GP morbidity surveillance network were used to investigate the distribution in the provision of STI care and the usefulness of GP data in surveillance.

**Methods:**

Data on STI-related episodes and STI diagnoses based on ICPC codes and, for chlamydia, prescriptions, were obtained from GP electronic medical records (EMRs) of the GP network and compared to data from STI centers from 2002 to 2007. Incidence rates were estimated for the total population in the Netherlands.

**Results:**

The incidence of STI-consultations and -diagnoses increased substantially in recent years, both at GPs and STI centers. The increase in consultations was larger than the increase in diagnoses; Chlamydia incidence rose especially at STI centers. GPs were responsible for 70% of STI-related episodes and 80-85% of STI diagnoses. STI centers attract relatively younger and more often male STI-patients than GPs. Symptomatic STIs like *Herpes genitalis *and genital warts were more frequently diagnosed at GPs and chlamydia, gonorrhea and syphilis at STI centers.

**Conclusions:**

GPs fulfill an important role in STI care, complementary to STI centers. Case definitions of STI could be improved, particularly by including laboratory results in EMRs. The contribution of primary care is often overlooked in STI health care. Including estimates from GP EMRs can improve the surveillance of STIs.

## Background

In the Netherlands, healthcare for people with sexually transmitted infections (STIs) is provided by general practitioners (GPs), specific STI centers, HIV treatment centers and specialized hospital care [1]. Patients will be referred from GPs or STI centers to receive more specialized care in HIV treatment centers or hospitals. In general, GPs act as 'gatekeeper', the primary point of access to healthcare [2]. The Netherlands accommodates over 4,300 general practices with about 8,700 practitioners and every Dutch inhabitant is connected to a general practice [3]. Costs of GP consultation, STI-tests and treatment are covered by the national -since 2006- statutory, health insurance [4]. STI care through specialized STI centers is an additional first line service, providing free, anonymous, low-threshold STI care to higher risk groups, fitting certain criteria (a.o. age, sexual preference, recent risk), who might not visit a GP for these specific problems. The STI centers receive government funding; the number of consultations in these facilities continuously increased in the past decade [5].

Reportedly, the majority of patients with STI-related problems are seen in primary care. In 2001, a survey in 75 practices estimated that countrywide 98,000 episodes at GPs were related to STI/HIV care [6], while STI centers reported 38,000 clients. In population surveys about sexual healthseeking behavior, the majority of persons with STI-related complaints or in want of an STI-test consulted a GP and of the remaining most went to an STI centre [7,8]. Of young people (under 25 years), also nearly three quarters said they would visit a GP if suspecting an STI [9]. Other health providers (i.e. hospital policlinics, centers for sexual health) have a limited role in STI care in the country. A minority of people looked for testing and treatment options via the internet. In other countries, such as Germany and the UK [10-12], specialized STI-clinics or other care facilities often play a more important role than GPs in STI-care.

In the Netherlands no complete case-registration exists for STIs, except for hepatitis B which is a notifiable disease. National surveillance of STI/HIV now focuses on STI centers and HIV-treatment centers [5]. thus obtaining detailed data from (presumably) high-risk populations. Although this surveillance is excellent to monitor trends and (re-) emergence of STIs, it obviously lacks data from the general population. With increasing numbers of clients in STI centers but limited insight in STI trends in general practices, the question arises whether this STI-service is starting to replace primary care.

General practitioners in the Netherlands, together with Sweden and Denmark, were ahead of other countries in using computers in their daily practice: 88% used electronic medical recording systems in 2001 [13]. Recently a new strategy for processing, analyzing and interpreting EMRs was developed within a national GP network [14] and estimates of yearly incidence rates became available for a range of health problems [15] defined by International Classification of Primary Care (ICPC) codes [16].

Here, we investigated the usefulness of EMRs from this national GP information network to fill the gap in STI surveillance in the Netherlands. Furthermore, we compared recent STI-trends and population characteristics of patients seen by GPs and STI centers, to evaluate whether these two complement each other in the provision of STI care.

## Methods

Data on STI diagnoses were obtained from the GPs EMR database of the Netherlands 'Information Network of General Practice' (LINH) and the national STI surveillance database of STI centers from the National Institute of Public Health (SOAP) from 2002 to 2007.

### General Practioners

#### Data collection

Anonymous GP data were obtained from the Netherlands Information Network of General Practice (LINH) after approval by the LINH steering committee. LINH started in 1992 and developed into a system recording longitudinal data on morbidity, prescriptions and referrals from patients subscribed in participating practices. The pool of practices fluctuates from year to year, but its composition is guarded to provide a representative sample of about 2% of the Dutch population regarding age and gender in comparison with Dutch National Statistics [17] and the practices are representative of all Dutch general practices with respect to geographical distribution and degree of urbanization [14,18,19]. For estimating morbidity rates, EMRs of individual GP-patient-contacts are grouped into episodes of care (concerning one health problem), as registered directly by the GPs (when available) or by constructing episodes with the validated application EPICON [20], grouping contacts with similar ICPC-codes less than two months apart. Individual patients' characteristics include age, sex, urban/rural residence (address density) and living in deprived areas (defined by GP-capitation fee).

#### Definition of STI diagnoses

In LINH, diagnoses are coded using International Classification of Primary Care (ICPC-1); laboratory results are unavailable within this registration system. In the majority of cases, GPs make the definitive diagnosis of the episode based on microbiological diagnostics [6]. We defined STI diagnoses as episodes registered with the STI-specific codes for men and women shown in Table 1. Episodes which the GP indicated with ICPC codes 'concern or fear of STI or HIV/AIDS' were considered as STI-related consultations.

**Table 1:** Case-definitions of STIs

	LINH (GP network)-ICPC* codes		*SOAP^† ^(STI centers)*	
	Men	Women	Men	Women
*STI-positive episodes*				
HIV infection/AIDS	B90		HIV-test	HIV-test
Non-specific urethritis	U72	*Not included*	Symptoms and exclusion of other STI	*Not included*
Syphilis	Y70	X70	Syphilis test	Syphilis test
Gonorrhea	Y71	X71	Gonorrhea test	Gonorrhea test
Trichomoniasis	*Not included*	X73	*Not included*	Trichomonas test
Herpes genitalis	Y72	X90	Symptoms, confirmed by test	Symptoms, confirmed by test
Genital warts	Y76	X91	Symptoms	Symptoms
Chlamydia				
- main codes‡	Y74, Y99	X84, X85, X74	Chlamydia test	Chlamydia test
- sub codes	Y99.3	X84.1, X85.1, X74.1		
*STI-related episodes*				
Fear of HV/AIDS	B25	B25	All consultations without one of the above STI-diagnoses	
Fear of STI	Y25	X23		

* ICPC = International Classification of Primary Care

† SOAP = National Surveillance of STI centers

‡ Chlamydia defined by main codes combined with prescriptions of antibiotics with following ATC-codes: J01FA10 J01AA02 J01CA04, J01CR02 J01FA01 J01MA01 or J01MA02

Chlamydia has no specific code in ICPC-1 but is recorded with codes for vaginitis (X84), cervicitis (X85) or PID (X74) in women and orchitis/epididimytis (Y74) or 'other genital diseases' (Y99) in men. Specific subcodes for chlamydia diagnoses (X84.1, X85.1, X74.1, Y99.3); exist within these main codes, but are not routinely used (i.e. GPs often only register main codes). For our analyses, we based the definition of chlamydia-diagnosis on a combination of code and prescription: episodes with subcodes were all counted as 'chlamydia', while episodes with main codes were included only when linked to chlamydia-related prescriptions Azythromycin, Doxycyclin, Amoxicillin, Erythromycin, Ciprofloxacin and Ofloxacin (coded by Anatomical Therapeutic Chemical Classification System; see Table 1).

#### Data analyses

Trends in incidence rates in LINH were calculated using multivariate multilevel repeated models for count data. Poisson multilevel regression analyses were conducted with three-level hierarchical structured data (patient, general practice as cluster-variable and year of recording), using MLWin 2.02 software. Not all practices could be included because of differences in electronic registration systems (70-75% of practices per year). The number of STI episodes was the outcome variable. The model was adjusted for patients age- and gender and the practice's length of recording [14]. For HIV, syphilis, gonorrhea, Trichomoniasis, NSU and 'fear of AIDS' counts were too low and therefore adjusted models were made without age/gender correction. Models were used to test linear trends over time. Chlamydia prevalence estimates were derived from the proportion of episodes with specified ICPC diagnoses and medication, calculated with data from 2006 and 2007. The total number of STI-related episodes at GPs was calculated by extrapolating to the Dutch population.

### STI centers

#### Data collection

The national STI surveillance based on data from STI centers consists of records of consultations in a real-time online database (SOAP), of clients' demographic characteristics, STI history, sexual behavior, STI tests and diagnosis. This collective anonymous database is managed at the RIVM for the purpose of national surveillance. Until 2002 this was based on voluntary registration of STI centers, which grew into a sentinel surveillance system of main centers in 2003 (80% coverage). Since 2004, all existing STI centers (n = 32) are connected, hence providing complete national coverage. The unit of reporting is 'client-visit'; repeated consultations from one client cannot be linked because of anonymousness.

#### Definition of STI diagnoses

In STI centers all STI diagnoses are laboratory-confirmed. Clients are routinely tested for chlamydia, gonorrhea and syphilis and the majority is also tested for HIV; Hepatitis-B, Trichomoniasis and *Herpes genitalis *are tested on indication; genital warts are reported optionally. Non-specific urethritis (NSU) is diagnosed based on symptoms, leucocyturia and exclusion of other diagnoses (Table 1).

#### Data analyses

STI reporting rates from SOAP were derived from real counts of STI diagnoses seen at STI centers per year, divided by the total Dutch population, based on the assumption that the 32 centers have national coverage although access to free testing is restricted to specific groups. No correction was made for data from 2002-2003 when national coverage was not yet achieved, but roughly estimated at about 80%. SPSS and SAS software was used for analyses.

## Results

From 2002 to 2007, 14,837 patient-episodes with ICPC codes for STI or 'fear of STI/HIV' were registered in the LINH GP surveillance network, which amounted to 0.4% of all episodes recorded, about 40 per practice per year (23 per fulltime-equivalent). At STI centers 352,524 client-visits were recorded, ranging from 200 per year in small (sub)centers to 26,000 in the largest clinic in Amsterdam.

### STI-trends

**Figure 1:**
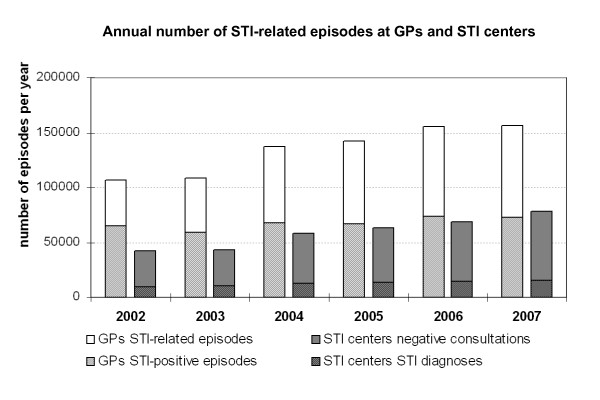
**Annual number of STI episodes at GPs (divided in STI-related episodes**, i.e. fear of STI/HIV, and episodes with STI/HIV diagnoses) and **consultations at STI centers**, divided in negative consultations (without diagnosis) and positive STI/HIV diagnoses, LINH-DB and SOAP surveillance, the Netherlands, 2002-2007.

There was a steady rise in demand for STI care over the period 2002-2007. At GPs, the incidence of STI-related episodes (STI + fear of STI/HIV) increased by 44%, from 664 per 100,000 patients in 2002 to 955/100,000 in 2007, equivalent to about 110,000 to 160,000 episodes countrywide. In this same period the number of client-visits at STI centers increased by 86% from 42,000 to 78,000 (263 to 488 per 100,000 inhabitants), more than expected due to 20% expansion of the centers under surveillance. The incidence of STI-positive cases (diagnosed with HIV, chlamydia, gonorrhea, syphilis, genital warts, *Herpes genitalis*, NSU or Trichomoniasis) increased by 20% at GPs and by 30% at STI centers from 2002 to 2007. At both facilities, the number of patients with STI-related negative episodes doubled (see Figure 1).

The ratio between STI-related episodes at GPs and STI centers decreased from 2.6:1 in 2002 to 2.0:1 in 2007. In 2007, two third of STI-related episodes was seen by GPs and one third by STI centers. Of all STI-positive episodes, 83% was recorded at GPs and 17% at STI-centers (81% of male and 85% of female STI-cases at GPs).

### Population differences

Visitors at STI centers were on average younger (30 years ± 0.04 SEM) than STI-patients at GPs (32 years ± 0.22 SEM, data 2007). The proportion of people under 25 years at STI centers was 40% and at GPs 35%; for women this was 54% versus 41%; 50-plussers represented 9% of STI patients at GPs and 5% at STI centers. The proportion of male clients was higher at STI centers than at GPs: 51% of visitors at STI centers were male and 44% of GP-patients with STI-related episodes were male.

About one third (38%) of the LINH-patient-population lived in highly urbanized areas; 9.5% lived in deprived areas. Patients with STI-related problems were more frequently from highly urbanized areas (62%); 17.6% lived in deprived areas. The client-population at STI centers could not be characterized in this way, but is assumed to be predominantly urban, because most STI centers are located in cities and the majority is seen at centers in the four largest Dutch cities (Amsterdam, Rotterdam, The Hague, Utrecht). At STI centers, specific high-risk groups were men who have sex with men (MSM, 28% visitors in 2007), female sex workers (9% of women) and clients of sex workers (9% of men).

### Chlamydia episodes at GPs

In a separate questionnaire to GPs participating in LINH (in 2007), the majority of GPs indicated to use sub codes for chlamydia. In 2006 and 2007, 454 episodes with chlamydia-specific ICPC sub codes were recorded. In addition, out of 4990 episodes with chlamydia-related main codes, 601 could be linked to chlamydia-prescriptions. This resulted in 1055 episodes of chlamydia by our case-definition. Five hundred sixty four episodes (53%) were in women, of which 26% was recorded with ICPC code X74 (PID), 47% with X84 (vaginitis) and 27% with X85 (cervicitis). In men, 41% of chlamydia episodes were recorded under Y74 (orchitis/epididimytis) and 59% under Y99 (other genital disease including chlamydia subcode). The proportion of chlamydia per ICPC code was 57% of X74 (PID), 8% of X84 (vaginitis), 51% of X85 (cervicitis), 32% of Y74 (orchitis/epididymitis) and 39% of Y99 (other genital diseases men).

Based on these results, the estimated reporting rate of chlamydia in 2006 and 2007 was 174 per 100.000. Chlamydia episodes were equally diagnosed in women (186/100,000) and in men (162/100,000). Of chlamydia patients under 25 years 83% were female. Male chlamydia patients were over-represented in the group of 50 years and older (81%).

### Trends in STI-diagnoses 2002-2007

**Figure 2:**
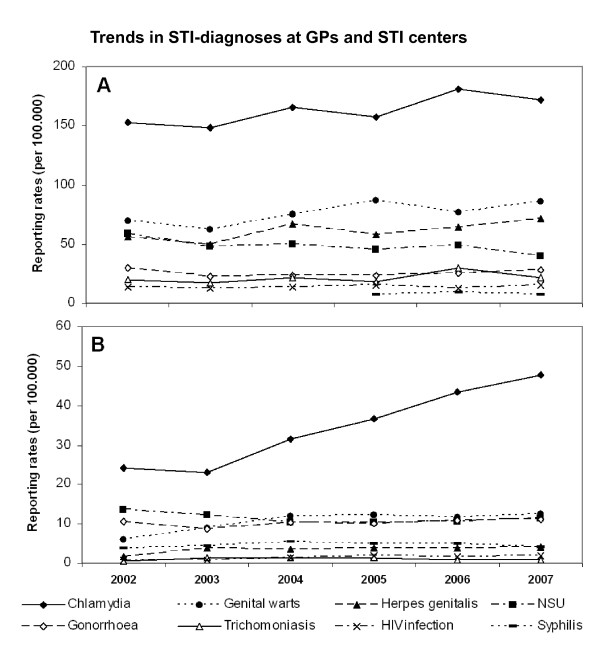
**Trends in STI-diagnoses registered by GPs (A) and STI centers (B) in the Netherlands, 2002-2007**. Estimates from GP surveillance (LINH-DB) extrapolated of 60 sentinel surgeries and numbers from national surveillance of 32 STI centers (SOAP).

**Figure 3:**
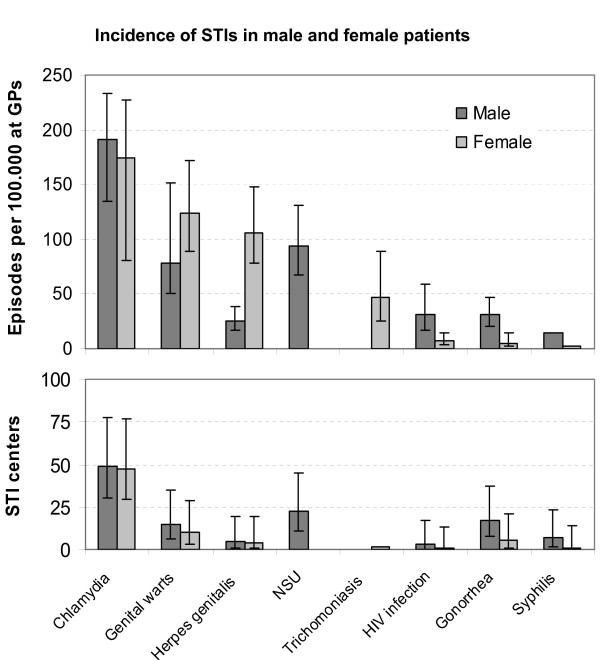
**Incidence of STIs in male and female patients seen at GPs (LINH-DB 2006) and at STI centers (SOAP 2007)**. Bars indicate 95% confidence intervals.

The most common STI at GPs was chlamydia, followed by genital warts and *Herpes genitalis *(Figures 2A and 3A). There was a significant linear increase in case-reports of chlamydia (in men and women) as well as genital warts and *Herpes genitalis *(in women), whereas rates of other STI infections remained relatively stable. Reporting rates for syphilis, gonorrhea and HIV at GPs were lower than the other STI; for syphilis no GP data were available for 2002-2004.

Chlamydia was also the most commonly reported STI at STI centers (Figures 2B and 3B) and its reporting rate rose sharply, in par with the rise in number of visitors to STI centers. Other STIs had quite stable reporting rates. Chlamydia, gonorrhea and syphilis were relatively more frequent diagnoses at STI centers, whereas Trichomoniasis, urethritis, *Herpes genitalis *and genital warts were more frequently recorded in general practices. HIV incidence was relatively high at GPs when compared to the STI centers. Chlamydia was reported at similar rates in men and women, both at GPs and at STI centers. Genital warts and *Herpes genitalis *were more often recorded in women but gonorrhea, syphilis and HIV more in men (Figure 3).

## Discussion

The STI healthcare seeking behavior in the Netherlands has increased considerably in recent years, both at GPs and STI centers. The rise was mainly due to a growing demand for testing and concern for STIs and to a lesser extent to increased STI-diagnoses. The total estimate at STI centers and at GPs equaled to an incidence of 1443 STI-related episodes and 570 STI-diagnoses per 100,000 persons in 2007. The increase in the sexual health seeking behavior is encouraging: rising from 0.9 to 1.4 consultations per 100 persons per year and even 4.3 per 100 young people under 25 years in 2008. This resulted in more STI diagnosis in recent years, especially of Chlamydia. About 70% of STI-related episodes were seen by GPs and 30% at STI centers; GPs accounted for 80-85% of positive STI-diagnoses. The incidence of chlamydia diagnoses increased strongly in STI centers and, to a lesser extent, at GPs. This reflects to a large extent the increase in testing volume; the positivity rate at the STI centers changed only moderately (from 9.2% in 2001 to 10.8% in 2008) [5]. Chlamydia case-reports in the two facilities together amounted to an estimated 36,000 cases per year in the Netherlands. STI centers saw relatively more young people and more male clients than GPs. Patients at STI centers were more at risk for STIs such as gonorrhea and syphilis, whereas GPs received more patients with genital warts, *Herpes genitalis*, urethritis and Trichomoniasis. STI centers fulfilled part of the growing demand for STI care, but did not 'take over' from GPs, who clearly remain the major firstline providers of STI care in the Netherlands. Both facilities saw rising numbers, indicating improved access to STI testing and -care and/or better awareness in the population.

### Limitations

Our analysis has several limitations due to the nature of the data sources.

Unfortunately laboratory results were not retrievable from the GPs EMR database, which makes unequal comparison to diagnoses in STI-centers. GPs nevertheless use laboratory tests to determine the final diagnosis of STI episodes in accordance with Dutch Guidelines for Primary Care, but we could not exclude misclassification. We recommend laboratory results to be included within future data extracts.

A second constraint was the absence of a specific ICPC code for chlamydia at GPs. Chlamydia can be reported with a sub-code in the ICPC classification, but most GPs register only main codes. Therefore we included chlamydia medication in our search strategy. This search strategy might induce a certain degree of overestimation. We found relatively high incidences in elderly men, where most genital infections are probably not STI related. Furthermore, chlamydia cases in women included diagnoses with codes for PID and also vaginitis, which is normally not caused by chlamydia, but controversially, a subcode 'vaginitis by chlamydia' exists in ICPC. Chlamydia, as the most prevalent STI, should have its own ICPC code.

For low-incident infections like syphilis and HIV, better recording at GPs is needed. In contrast to chlamydia and gonorrhea infections these patients are often referred to secondary level. Due to recording of cases as 'new HIV infection' at the GP which may already have been reported elsewhere, substantial double counting may arise. The annual number of HIV-infections based on data presented here would be about 2500 in recent years, whereas current figures of HIV-monitoring show only around 1000 new cases per year are admitted to HIV-treatment centers [5]. More information on the registration of HIV-infections at GPs is desirable, because of uncertainties about the -presumably substantial- number of HIV-positives unaware of their status [21].

### Discussion of findings

Differences in population characteristics of patients at GPs and STI centers suggest that each facility serves a different target-group. Clients at STI centers seem to come more often for a preventive STI-check-up (without obvious symptoms) and GP patients with commonly symptomatic STIs. The threshold to go for an (anonymous) STI-check is low at STI centers offering free testing to high-risk groups. The proportion of MSM at STI centers (28% of men) was much higher than 3-8% estimated in the general population [7] and among GP patients [22]. GP patients are probably less often from high-risk groups [23] than in STI centers, however we saw that a substantial group came from highly urbanized or deprived areas. To determine to what extent specific STI risk-groups also come to GPs, more background information, such as sexual preference or reason for consultation, is needed.

Compared to other countries, GPs in the Netherlands play a large role in STI care. In Belgium, the major part of STIs is also detected by GPs, since most of the population at risk has access to this setting only for STI care [24]. In Germany, data from sentinel surveillance suggest that private practitioners see smaller numbers of STI-patients than hospital-based STI clinics or local health offices; their patients are less often from high-risk groups [10]. In the UK, the majority of STI diagnoses are made at Genitourinary Medicine (GUM) clinics, though a substantial proportion of chlamydia cases in females is diagnosed at GPs [11,12]. In the US STI care is spread over different facilities [25]. In Australia, a cross sectional survey among GPs showed that GPs are confronted with STI-related problems regularly, although the STI diagnosis often remained unconfirmed [26,27].

The incidence of STI-related episodes we found (1400/100,000) is quite similar to reports of 1.2% of adults with self-reported signs and symptoms suggestive of an STI [7], but lower than the reported 4% of adults who had an STI/HIV check-up [8]. The ratio of consultations at GPs versus STI centers is in line with earlier data [5,7,8,28]. The 36,000 chlamydia cases we estimated per year is lower than the 60,000 per year estimated in a chlamydia pilot screening [29], but the difference can be explained by the proportion asymptomatics, which only come forward in a screening program.

A comparison with other European countries shows that the incidence rates we estimated for chlamydia (220/100,000, from GP + STI centers together), gonorrhea (39/100,000) and syphilis (11/100,000) are higher than overall averages for Europe reported by ECDC: chlamydia 92/100,000, gonorrhea 9/100,000 and syphilis 4/100,000 [30]. This may be due to actual differences in incidence, or the relatively easy access to STI-care in the Netherlands and related testing-rates, but also to completeness of reporting. Current STI surveillance systems across Europe show a large heterogeneity [10,31]. Data are more complete in northern European countries, where they are based on comprehensive case reports from diverse medical care facilities (Scandinavian countries) or main STI-care providers (GUM clinics, UK) [32]. Our incidence rates for chlamydia and gonorrhea are in line with rates reported from these northern European countries to ESSTI [32], but our syphilis rates were higher than theirs. The incidence rates in the United States, where these three STIs are notifiable diseases, are higher for chlamydia (370/100.000) than in the Netherlands but comparable to some other northern European countries; gonorrhea incidence is also higher in the US while syphilis rates in the US are similar to European levels [25]. Incidence rates for chlamydia in the Netherlands were similar in men and women, whereas in most other northern European countries incidence rates in women are higher [32]. In the UK the male:female ratio is balanced as well [32]. In the US chlamydia incidence is three times higher in women [25]. Differences may be explained by access to care and STI-testing habits.

## Conclusion

In conclusion, the addition of data from GP surveillance will improve the completeness of surveillance of the main STIs in the Netherlands and facilitate international comparisons. For low-prevalent STIs such as syphilis and HIV, data from the GP network may be limited. GPs still attend to the majority of STI patients in the Netherlands even though the popularity of publicly funded STI centers increased.

## Competing interests

The authors declare that they have no competing interests.

## Authors' contributions

IB designed the study, interpreted the data and drafted the manuscript. RV coordinates the GP network and its data acquisition, advised and commented on earlier drafts of the manuscript. CD calculated trends for STI episodes in the GP network while FK contributed to the analyses of data from the STI centers. MS and JB helped with conception and design of the study and revised the manuscript critically. All authors read and approved the final manuscript.

## Pre-publication history

The pre-publication history for this paper can be accessed here:

http://www.biomedcentral.com/1471-2296/11/39/prepub
